# 

**Published:** 1916-02

**Authors:** 


### Contents

Event and Comment .... 51
The Shortage of Platinum .... 55
Efficiency in Tooth Brushing .... 60
Fixed Laws Governing Dental Amalgams .... 62
Diagnostic Methods for Anesthesia .... 67
The Dentist and the Orthodontist .... 71
Some Refractories Used in Dentistry .... 75
Succinimid of Mercury .... 85
Indents .... 89
Bibliography .... 98
Announcements .... 99

